# Optical Intensity Discrimination with Engineered Interface States in Topological Photonic Crystals

**DOI:** 10.3390/mi17020165

**Published:** 2026-01-27

**Authors:** Bartosz Janaszek, Paweł Szczepański

**Affiliations:** 1 Institute of Microelectronics and Optoelectronics, Warsaw University of Technology, Koszykowa 75, 00-665 Warsaw, Poland; 2 National Institute of Telecommunications, 1 Szachowa Str., 04-894 Warsaw, Poland

**Keywords:** Kerr effect, topological states, photonic crystals, optical coatings, nonlinear DBR

## Abstract

We propose a 1D photonic crystal with nonlinear graphene–spacer–graphene truncation, which enables a tunable, non-monotonic, and intensity-dependent transmission response. By employing synthetic geometrical space to obtain Fermi arc states, the structure is designed to support a real-space topologically protected Tamm plasmon polariton, revealing an intensity-dependent transmission peak within the THz spectral range. As such, the proposed thin-film structure may serve as a nonlinear DBR element that can be integrated into a laser cavity to provide intensity-selective feedback, thereby facilitating controllable pulse shaping and enabling passive pulse formation mechanisms such as mode-locking or Q-switching. Due to its topological robustness, spectral scalability, and electrical tunability via graphene biasing, the platform provides a new route toward compact, reconfigurable nonlinear reflectors for efficient and controllable laser pulse generation, thereby extending the functionality of conventional saturable absorbers and semiconductor DBRs.

## 1. Introduction

Photonic structures that combine strong light–matter interaction, engineered dispersion, and nonlinearity are pivotal to contemporary photonics. The optical Tamm plasmon polariton (TPP), i.e., an interface state existing between a one-dimensional (1D) photonic crystal and a reflective layer, exhibits a narrowband and strongly confined electromagnetic mode that can be excited at normal incidence and tailored in frequency and linewidth by design. Different architectures have been demonstrated in the visible-to-THz bands and are actively pursued for sensing [1,2], spatial control of thermal radiation [3], narrowband absorption [4], and enhanced nonlinear effects [5,6]. More recently, topological valley photonics has emerged as a promising approach to compact, low-loss terahertz (THz) topological devices. By exploiting valley-dependent interface states, this approach enables robust waveguiding and cavity formation with strongly suppressed radiation losses. In particular, recent demonstrations of ultrahigh-Q topological cavities and waveguides in the THz regime highlight the feasibility of low-loss, integrated topological THz photonics [7,8].

Graphene is particularly suitable for THz TPP devices due to its strong, tunable plasmonic response, as well as its strong Kerr nonlinearity [9]. Graphene-based multilayer and spacer geometries have emerged as highly efficient THz absorbers [10], compact diffraction-free lensing platforms [11], and miniaturized graphene antennas capable of dynamic amplitude and phase manipulation [12]. Beyond linear optical components, recent progress in graphene nonlinear photonics has demonstrated that the exceptional nonlinearity of graphene can be harnessed to achieve a range of intensity-dependent THz functionalities. Until now, it has been demonstrated that graphene–spacer–graphene truncations embedded in resonant structures have been shown to substantially enhance third-harmonic generation in the THz regime [13,14]. Furthermore, coherent THz generation exploiting nonlinear carrier dynamics under strong optical or THz pumping has been experimentally confirmed, illustrating the potential of graphene for compact, electrically tunable THz sources [15].

At the same time, topological photonics and the emerging use of synthetic spaces to emulate topological phenomena [16,17] have expanded the robustness available of planar devices. Synthetic geometrical parameter spaces allow us to map 1D or 2D photonic structures onto higher-dimensional topological models and to access phenomena normally associated with 3D Weyl/semimetal physics [16]. Photonic realizations of Fermi–arc–like surface states and the controlled reconstruction of those states in engineered 1D platforms have been reported in recent years [18,19], opening a pathway to surface modes that are robust against certain classes of disorder and that show exotic dispersion engineering possibilities.

Combining these threads, i.e., Tamm plasmon polaritons, graphene’s THz nonlinearity, and engineered topological protection via synthetic dimensions, creates a compelling platform for surface states of controllable nonlinear properties, which we utilize to achieve an intensity-dependent and non-monotonic transmission with a tunable transmission maximum at a target intensity. For this purpose, we propose a 1D photonic structure that emulates a Weyl point within the THz spectral range. To simultaneously achieve high sensitivity to light intensity changes and strong reflection, we apply truncation with two graphene sheets (pristine and externally electrically biased) separated with a subwavelength dielectric spacer. It is worth underlining that the proposed architecture, in contrast to similar solutions proposed previously and based dominantly on saturable absorption [16,17] or optical pumping of materials with optical gain [20], relies on Kerr nonlinear and self-induced changes in reflection, which allows for minimizing optical absorption and enhancing thermal stability, without any external steering factor. As such, the proposed device can be effectively applied as an intensity discriminator coating for a lens or mirror, revealing optical power-limiting performance that can be employed to enable new passive pulse formation mechanisms in a laser cavity.

## 2. Numerical Simulation

In this section, we outline the key assumptions underlying our numerical calculations, which enable the replication of the presented analysis. In particular, this section contains (i) an assumed numerical model of graphene accounting for nonlinear and linear properties and (ii) the description of the considered photonic crystal revealing a Weyl point.

### 2.1. Nonlinear and Linear Properties of Graphene

In this work, we utilize the nonlinear and plasmonic properties of graphene to achieve intensity-dependent behavior of topological surface states. For that purpose, graphene is modeled as an infinitesimally thin sheet located at the truncation plane of a 1D PC with conductivity calculated as a sum of intraband and interband contributions, with Kerr-type nonlinearity retained for the intraband term [21]:

(1) $$\sigma \left(\omega ,\mu_{C},A\right)=\sigma_{intra}^{L}+{\left|A\right|}^{2}\cdot \sigma_{intra}^{NL}+\sigma_{inter}^{L},$$

where A is the electric field amplitude in V/m units and, for linear terms, we employ the well-established Kubo/Drude form [22]:

(2) $$\sigma_{intra}^{L}=-j\frac{e^{2}k_{B}T}{\pi \hbar^{2}\left(\omega -2j/\tau \right)}\left(\frac{\mu_{c}}{k_{B}T}+2ln\left(e^{-\frac{\mu_{c}}{k_{B}T}}+1\right)\right),$$

(3) $$\sigma_{inter}^{L}=\frac{-je^{2}}{4\pi \hbar }ln\left(\frac{2\left|\mu_{c}\right|-\left(\omega -2j/\tau \right)\hbar }{2\left|\mu_{c}\right|+\left(\omega -2j/\tau \right)\hbar }\right),$$

where ω denotes the angular frequency of the incident electromagnetic wave, while $\mu_{c}$ represents the chemical potential of graphene. The calculations are performed at ambient temperature T = 300 K. Carrier scattering can be calculated from $\tau =\mu \cdot \mu_{C}/e\cdot v_{F}$ [23,24], where μ = 10,000 cm^2^/Vs is the mobility of charge carriers in graphene and $v_{F}$ = $10^{6}\;m/s$ is the Fermi velocity of graphene. The fundamental physical constants e, $k_{B}$, and ℏ correspond to the elementary charge, the Boltzmann constant, and the reduced Planck constant, respectively. It is worth to underline that those expressions are standard in graphene EM modeling and are widely used in THz photonics [15,25]. The nonlinear optical response of graphene in the intraband regime is incorporated through a third-order surface conductivity term. Under the condition ωτ≫1, which is satisfied in the frequency range considered, this term can be expressed as follows [23]:

(4) $$\sigma_{intra}^{NL}=-j\frac{3}{32}\frac{e^{2}}{\pi \hbar }\frac{{\left(ev_{F}\right)}^{2}}{\mu_{c}\omega^{3}},\;$$

for which the influence of two-photon absorption is neglected due to its experimentally proven low impact and magnitude [26]. It is worth noting that the negative imaginary part of this nonlinear term indicates that graphene exhibits a self-focusing Kerr-type nonlinear response in this regime with sub-picosecond intraband relaxation time [27]. In the context of considered application, the temporal response of the proposed structure is dominantly governed by the sub-picosecond intraband relaxation dynamics of graphene, while the response of the remaining constituents may be regarded as quasi-static on this timescale. Although pulse formation dynamics are additionally influenced by gain-medium transients and cavity effects, extensive experimental evidence indicates that graphene-based nonlinear elements are sufficiently fast to support real-time pulse action [28,29]. It is also worth to underline that the employed graphene’s nonlinear response model is consistent with theoretical predictions based on quasi-classical transport models of Dirac carriers [30]. In this spectral range, higher-order processes such as two-photon absorption are expected to be negligible [23]. The Kerr-type model represents a perturbative approximation and does not explicitly account for carrier heating or saturation effects, which may become relevant at sufficiently high field strengths. Nevertheless, within the operating regime considered here, it captures the dominant nonlinear contribution.

Additionally, the chemical potential of graphene $\mu_{c}$ may be tuned via external gate voltage $V_{g}$ applied between graphene and a gate electrode. Under the parallel-plate capacitor approximation and linear dispersion of graphene, the chemical potential is related to biasing voltage by the following relationship [31]:

(5) $$\left|\mu_{c}\right|=\hbar \upsilon_{F}\sqrt{\pi \left|a_{0}\left(V_{g}-V_{dirac}\right)\right|},$$

where $V_{dirac}$ = 10 mV, reflecting weak chemical doping (or impurity) of graphene sheets and satisfying the high frequency approximation of the conductivity intraband term, and $a_{0}=9\times 10^{16}\;m^{-1}V^{-1}$ being an empirical constant [32]. Finally, the permittivity of graphene can be calculated based on its total conductivity described in Equation (1) and respective terms expressed in Equations (2)–(4):

(6) $$\varepsilon_{g}(\omega ,\mu_{c},A)=1-j\frac{\sigma \left(\omega ,\mu_{C},A\right)}{\omega \varepsilon_{o}t_{g}},$$

where $t_{g}\;$= 0.35 nm is the thickness of graphene monolayer. It is worth noting that the wave amplitude can be converted to intensity as follows: $I_{inc}=c_{0}\cdot \varepsilon_{0}\cdot {\left|A\right|}^{2}/2$, with light speed in vacuum denoted as $c_{0}$ and electric permittivity of vacuum as $\varepsilon_{0}$, respectively.

**Figure 1:**
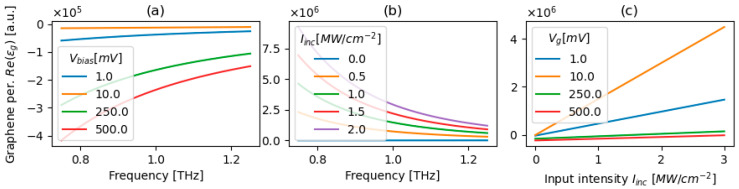
Spectral (**a**,**b**) and intensity-dependent characteristics (**c**) of graphene’s permittivity for various levels of biasing voltage and/or incident optical intensity.

Figure 1a–c illustrate how the permittivity of graphene, described with Equation (6), is affected by electrostatic biasing and optical intensity, highlighting a clear interplay between voltage control and nonlinear response, please refer to Equations (2)–(5) for related dependencies of graphene’s conductivity. This behavior demonstrates that the sensitivity of graphene’s permittivity to intensity decreases with increasing voltage bias. Physically, higher biasing increases the chemical potential and the linear intraband conductivity, which in turn diminishes the relative contribution of the nonlinear intraband term to the total response. As a result, graphene transitions from a strongly intensity-sensitive medium at low bias to a more voltage-dominated, weakly nonlinear regime at high bias.

### 2.2. Weyl Point and Optical Properties

**Figure 2:**
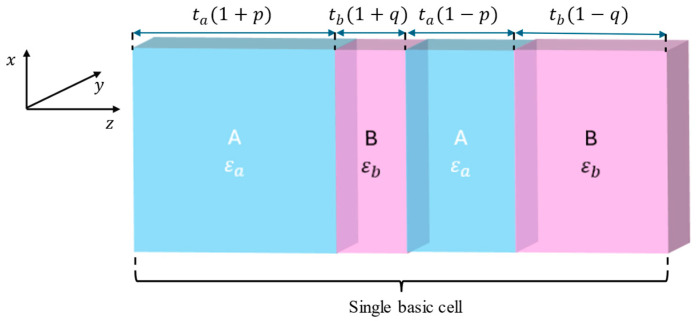
The basic cell of the proposed 1D photonic crystals. The thicknesses $t_{a}\;$= 22 µm and $t_{b\;}$= 14 µm have been selected to obtain the Weyl point located around 1 THz frequency.

Following the method of emulating Weyl point in a synthetic geometrical space [18,19], instead of 3D momentum space, which is typically employed to achieve Weyl point in photonics [16], we consider a synthetic geometrical space composed of component ($k_{z}$) and two independent geometric parameters p and q. Here, we design a 1D photonic crystal with parametrized 4-layer basic cells composed of materials A and B, described with $\varepsilon_{a}\;$= 5.76 and $\varepsilon_{b}\;$= 2.04 corresponding to optical properties of PTFE and fused silica at THz frequencies, respectively, and parametrized with two independent geometrical parameters, (see Figure 2). The assumed unitless parameters $p\in \left[-1,1\right]$ and $q\in \left[-1,1\right]$, in line with the approach proposed by Wang et al. [19], are applied to vary thickness of layers constituting the basic cell from fixed values of $t_{a}$ and $t_{b}$.

For a structure of such a basic cell, the dispersion properties can be obtained by solving the eigenvalue Bloch wave problem, which, according to Yeh’s approach and general assumptions of the transfer matrix method (TMM) [19,33], can be formulated from the components of the basic cell’s transfer matrix, which in turn results from the multiplication of matrices describing layers constituting the cell.

**Figure 3:**
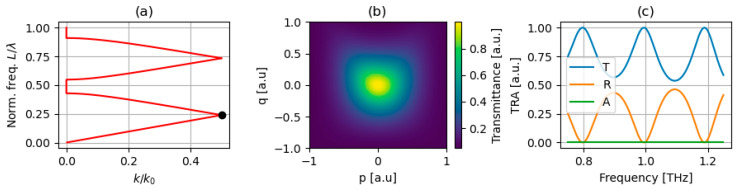
The band dispersion of the considered PC (**a**) with the considered Weyl point indicated with a black dot. Optical properties of the PC structure consisted of N = 5 basic cells plotted vs. geometrical parameters at the Weyl point frequency $f_{weyl}\approx 0.994\;THz$ (**b**), as well as spectral characteristics of transmittance, reflectance, and absorption at p = q = 0 (**c**).

In line with the assumed approach, the Weyl points can be observed as degeneracy points located at $k=k_{0}/2$ connecting lower and upper dispersion bands. In particular, we focus on the first-order Weyl point, which exists at the point where the first and second bands intersect (denoted with a black dot in Figure 3a) and corresponds to a specific frequency, referred to as the Weyl frequency. Then, for a given frequency and considered structure, power transmission (transmittance), reflection (reflectance), and absorption can be calculated by using the TMM formalism [18]. Figure 3b illustrates transmittance plotted against the geometrical parameter space, which is characterized by a large peak around p = q = 0 corresponding to one of the F-P resonance modes existing in the considered spectrum, (see Figure 3c). Now, to obtain a TPP state existing at the interface, the following resonance condition for reflection phase needs to be met:

(7) $$\varphi_{PC}+\varphi_{r}=2\pi m,$$

where $\varphi_{PC}$ and $\varphi_{r}$ are phase contributions from the photonic crystal and reflective layer truncating the structure, respectively. Therefore, conditions for the existence of TPP states can be obtained by application of almost any reflective layer, i.e., it can be expected there will be at least a single *pq* pair providing appropriate phase conditions.

## 3. Results and Discussion

**Figure 4:**
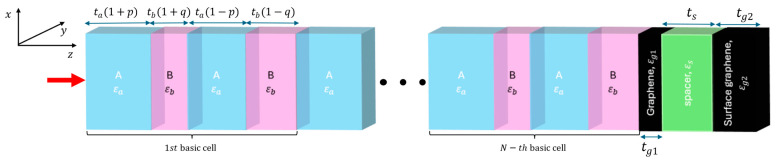
The schematic illustration of the considered PC truncated with graphene/spacer/graphene, creating an interface that supports a topological surface state. The chosen parameters are $t_{g1}\;$= 0.35, $t_{s}\;$= 1000, and $t_{g2}\;$= 1.05 nm. The red arrow indicates the propagation direction.

The primary objective of this work is to utilize topological surface states to achieve low-loss, intensity-dependent transmission. However, to provide conditions (see Equation (7)) for the existence of such a state at the interface, a strong reflection must be provided. As such, a strong reflection may be obtained by applying a graphene monolayer biased with a high voltage, which, on the other hand, will significantly lower sensitivity to optical intensity changes, as seen in Figure 1a–c. Thus, to overcome the limitations of this tradeoff, we propose a graphene/spacer/graphene truncation, consisting of an unbiased graphene monolayer ($V_{g1}\;$= 1 mV), dielectric spacer of thickness (described with permittivity $\varepsilon_{s}$), and tri-layer graphene biased with high voltage ($V_{g2}$ = 5 V), as seen in Figure 4. This configuration allows us to simultaneously achieve strong reflection providing conditions for the existence of Tamm plasmon polaritons and high sensitivity to the intensity change. It is worth to underline that the spacer thickness of the dielectric spacer has a negligible impact on the resulting nonlinear performance and was primarily chosen to provide electrical isolation between the graphene sheets, thereby enabling separation of their electronic bands. Furthermore, the spacer thickness was intentionally kept much smaller than the operating wavelength to suppress additional interference effects, which could otherwise obscure the physical effects associated with the presence of the Tamm plasmon polariton. We note that further systematic optimization of the spacer thickness and graphene layer configuration, under application-specific criteria such as required incident power, voltage biasing constraints, or technological limitations, could lead to improved performance. It is worth to underline that the proposed structure is fully planar and composed of a periodic arrangement of PTFE and fused silica layers with micrometer-scale thicknesses, which can be fabricated using established thin-film deposition, bonding, or lamination techniques [34,35]. The truncation consists of graphene sheets separated by a 1 µm silica spacer, a configuration compatible with standard graphene transfer procedures that have been widely demonstrated experimentally [36]. Since the design does not require lateral patterning or complex three-dimensional structuring, it is well suited for experimental realization using current fabrication capabilities.

**Figure 5:**
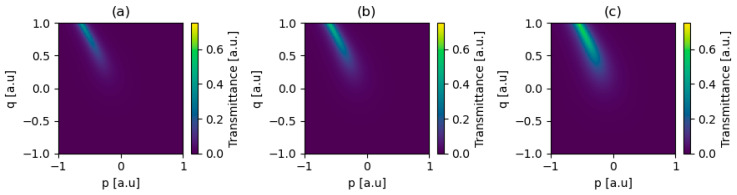
Transmittance for the PC structure with graphene/space/graphene truncation for various incident optical intensities: 0 (**a**), 0.5 (**b**), and 1 MW/cm^2^ (**c**).

For the proposed truncation geometry, we calculate transmittance colormaps as functions of the geometrical parameters p and q, as shown in Figure 5a–c. Each colormap is evaluated at the calculated Weyl frequency and for different incident intensities, i.e., 0, 0.5 and 1 MW/cm^2^. For a given intensity, all pq pairs satisfying resonance conditions given by Equation (7) form a continuous arc-like shape in parameter space, which is the reason of commonly reference to those modes as Fermi arc states.

With increasing incident intensity, both the maximum transmittance and the shape of the arc undergo slight modifications. This behavior originates from intensity-induced changes in the phase of the reflected waves, resulting from the Kerr-type nonlinear response of graphene. Notably, this effect can be exploited to engineer a non-monotonic transmission characteristic. Specifically, by selecting the pq pair yielding the highest transmittance at a targeted intensity, the structure can be designed to exhibit a transmission peak at that intensity.

**Figure 6:**
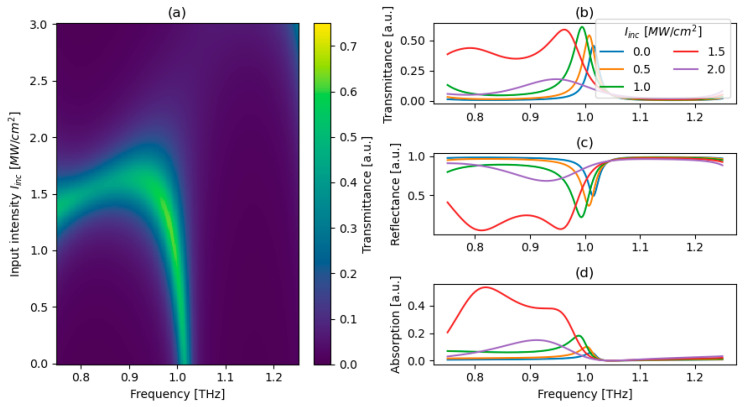
Transmittance colormap plotted as a function of frequency and incident intensity (**a**). Transmittance (**b**), reflectance (**c**), and absorption (**d**) spectra for different incident intensities. Structure with parameters p = −0.53, q = 1.00 and composed of *N* = 5 basic cells, $V_{g1}$ = 1 mV.

As an example, we choose an incident intensity of 1 MW/cm^2^ for further analysis, for which the peak transmittance occurs at p = −0.53, q = 1.00. The detailed behavior of transmittance, reflectance, and absorption for the chosen pq pair evaluated as a function of THz frequencies and incident optical intensities is presented in Figure 6.

**Figure 7:**
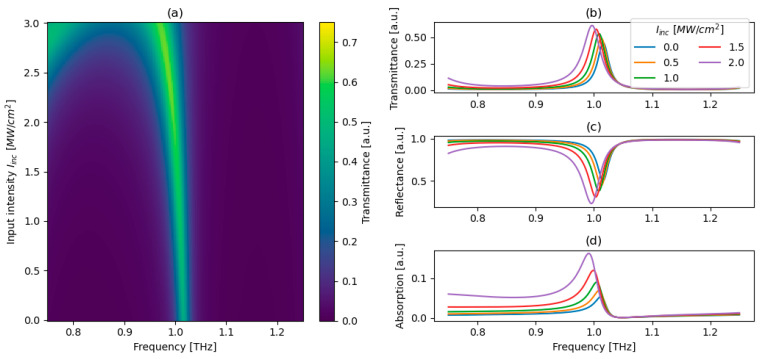
Transmittance colormap plotted as a function of frequency and incident intensity (**a**). Transmittance (**b**), reflectance (**c**), and absorption (**d**) spectra for different incident intensities. Structure with parameters p = −0.53, q = 1.00 and composed of *N* = 5 basic cells, $V_{g1}$ = 100 mV.

Figure 6a summarizes the nonlinear behavior of transmittance of the proposed structure and highlights its power-limiting behavior. At low intensities, a pronounced and well-defined transmission peak is observed, forming a transmission channel. As the incident intensity increases, the observed channel bends, ultimately exhibiting a clear intensity cut-off beyond which transmission is strongly suppressed. This abrupt reduction in transmission constitutes the core power-limiting mechanism of the proposed device and indicates a transition from a transmissive to a predominantly reflective regime, which can be confirmed with Figure 6b–d illustrating spectral cuts of transmittance, reflectance, and absorption at selected intensities, revealing the underlying redistribution of optical energy. Notably, at the bending point of the transmission channel, an enhanced and spectrally broadened absorption band emerges. This feature originates from broadband coupling between the incident field and the topologically protected TPP modes supported at the truncation interface, which may be connected to the dispersive nature of graphene’s nonlinear response, which is stronger at lower frequencies, as shown in Figure 1. As the intensity increases, the nonlinear refractive index of graphene grows, leading to an enhanced phase contribution at the graphene–dielectric interface. This intensity-dependent phase shift enables the resonance condition from Equation (7) to be satisfied at longer wavelengths, resulting in the observed channel bending. It is worth noting that the absorption-induced thermal effects may, in principle, influence the long-term stability of the proposed structure if the damage thresholds of the constituent materials are exceeded. In the current configuration, the absorption peak observed at the bending point of the transmission channel primarily originates from strong field localization at the graphene–dielectric interfaces. As a result, the dominant contribution to dissipation occurs in the vicinity of the graphene sheets, which have been reported to withstand optical power densities as high as 360 GW/cm^−2^ under comparable conditions [37]. Consequently, thermal degradation is not expected to constitute a critical limitation within the operational regime considered here. Moreover, the absorption level may be further reduced through appropriate optimization of graphene biasing or by employing materials with lower intrinsic losses. The cut-off threshold itself can also be actively tuned via electrostatic biasing of the unbiased graphene sheet ($V_{g1}$ = 1 mV). As presented in Section 2, increasing the bias voltage $V_{g1}$ reduces the effective nonlinear sensitivity of graphene, leading to a higher intensity required to trigger resonance detuning and transmission suppression, (see Figure 7). This voltage-controlled desensitization provides an external degree of freedom for dynamically adjusting the limiting threshold without modifying the photonic crystal geometry. Combined with the inherent frequency scalability of the structure, which is achievable through geometric rescaling, this feature enables a versatile, tunable, and broadband platform for optical power-limiting and protection of sensitive intracavity and downstream optical components across the THz spectral range.

**Figure 8:**
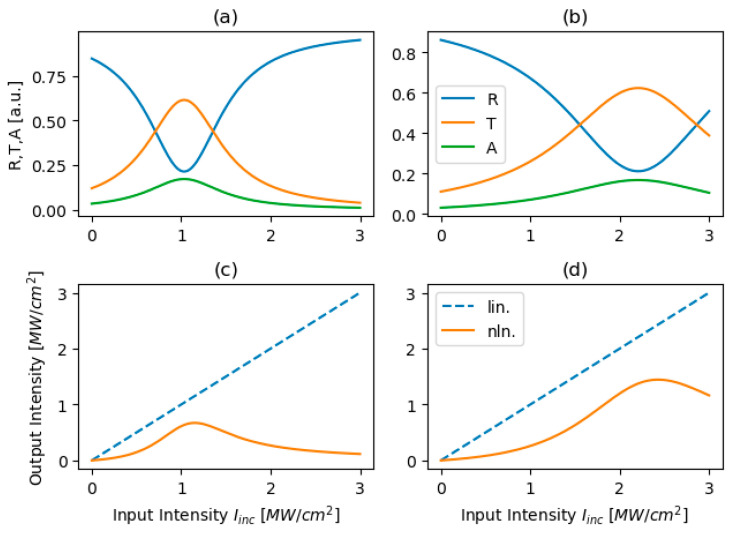
Relative (**a**,**b**)—i.e., reflectance, transmittance, and absorption coefficients—and absolute (**c**,**d**) power-limiting performance for PC structure biased with two different voltage biasing cases: $V_{g1}$ = 1 mV (**a**,**c**) and $V_{g1}$ = 100 mV (**b**,**d**).

Figure 8 illustrates the power-limiting performance of the proposed structure for two different graphene-biasing levels, highlighting its relevance as a nonlinear DBR. Panels (a) and (b) show the relative redistribution of optical power among reflectance, transmittance, and absorption as a function of incident intensity, revealing a pronounced non-monotonic transmission peak followed by a dominant reflective response at higher intensities, which may effectively suppress intracavity power build-up. Panels (c) and (d) present the corresponding input–output characteristics, where the linear response (dashed line) is contrasted with the nonlinear behavior (solid line). In both cases, the output intensity initially increases but then saturates and decreases beyond a certain intensity threshold. Increasing the graphene bias voltage shifts the onset of limiting toward higher input intensities, consistent with a reduced nonlinear sensitivity, thereby enabling external tuning of the threshold. In this regime, the structure operates as a bias-tunable, predominantly reflective nonlinear Bragg element that provides intensity-selective optical feedback. Such controllable reflectivity modulation supports the suppression of low-intensity continuous-wave components while favoring high-intensity pulse formation, offering a robust and thermally efficient pathway toward electrically adjustable mode-locking and pulse shaping in laser cavities.

## 4. Conclusions

We have introduced and analyzed a planar one-dimensional photonic crystal with a nonlinear graphene/spacer/graphene truncation that supports a topological Tamm plasmon polariton in the terahertz spectral range. By employing a synthetic geometrical space framework to substitute the three-dimensional momentum space, the proposed structure enables the emergence of topologically protected interface states whose spectral position and transmission characteristics can be dynamically controlled through optical intensity and electrical biasing of graphene. Additionally, the robustness against internal geometrical deviations has been assessed to complement the analysis and is included in the Appendix A.

The interplay between graphene’s dispersive nonlinearity and the topological nature of the interface state gives rise to a non-monotonic, intensity-dependent transmission response, enabling applications requiring power-limiting and intensity-selectivity. Owing to the ultrafast intraband carrier dynamics of graphene, the temporal response of the structure is expected to be sufficiently fast to support real-time pulse formation processes in laser cavities, while the ultimate pulse characteristics will be governed by the combined dynamics of the nonlinear interface and the gain medium. A comprehensive time-domain analysis of transient laser dynamics and thermal effects is identified as an important direction for future work.

Importantly, the proposed design relies exclusively on planar multilayer stacks of PTFE and fused silica of micrometer-scale thicknesses, combined with graphene sheets separated by a dielectric spacer, making it fully compatible with existing fabrication technologies. Together with its electrical tunability, spectral scalability, and intrinsic robustness (for details on robustness analysis, please see Appendix A), the proposed structure represents a compact and reconfigurable nonlinear distributed Bragg reflector that complements and extends the capabilities of conventional saturable absorbers and semiconductor DBRs.

Overall, this work establishes a versatile topological photonic platform for nonlinear THz optics and laser pulse control, opening new opportunities for robust, electrically tunable, and integration-ready nonlinear elements in advanced photonic and laser systems.

## Figures and Tables

**Figure A1:**
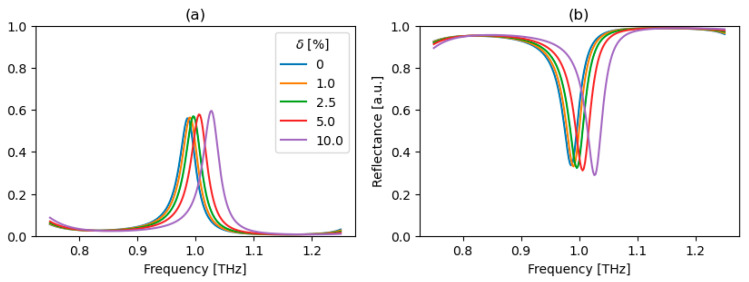
Transmittance (**a**) and reflectance (**b**) spectra for various values of the δ parameter. The intensity of incident light is $I=1\;\frac{MW}{{cm}^{2}}$.

**Figure A2:**
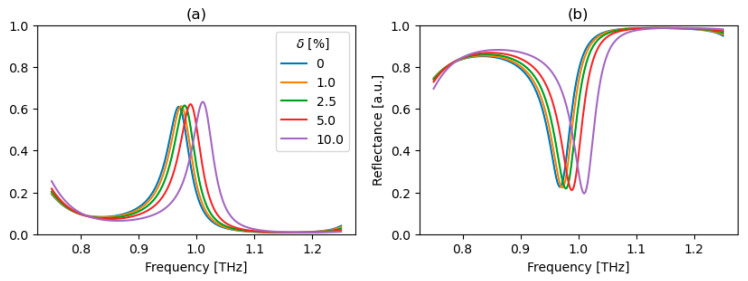
Transmittance (**a**) and reflectance (**b**) spectra for various values of the δ parameter. The intensity of incident light is $I=1.5\;\frac{MW}{{cm}^{2}}$.

**Figure A3:**
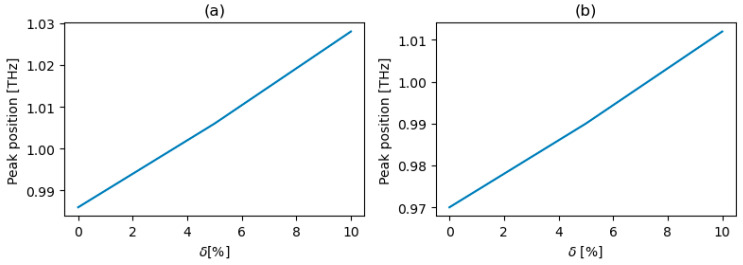
Resonance spectra shift plotted vs. δ parameter for the intensity of incident light is I= 1 (**a**) and 1.5 $\frac{MW}{{cm}^{2}}$ (**b**).

## Data Availability

The original contributions presented in this study are included in the article. Further inquiries can be directed to the corresponding author.

## Abbreviations

The following abbreviations are used in this manuscript:

TPP	Tamm Plasmon Polariton
TMM	Transfer Matrix Method
F-P	Fabry–Perot
PTFE	Polytetrafluoroethylene (Teflon)
DBR	Distributed Bragg Reflector

## Appendix A

Here, we present a supplementary analysis on the robustness of the proposed device towards errors that may arise during fabrication and deposition processes. For this purpose, we define a parameter δ with local fabrication errors of layer deposition by modifying thickness of each layer as follows: $t_{i}=t_{a(b)}\cdot \left(1\pm p\left(q\right)+\delta_{i}\right)$, where i is the number of layer and $\delta_{i}$ is a random number from uniform distribution of range $\delta_{i}\in \left(-\delta ,\delta \right)/100$ picked separately for each layer. To investigate the impact of possible fabrication errors, we calculated transmittance and reflectance spectra for various values of parameter δ (Figure A1 and Figure A2) as well as the related shift in the resonance frequency (Figure A3). The calculation has been performed for previously investigated structure parametrized with p = −0.53, q = 1.00, and composed of N = 5 basic cells. The considered structure is truncated with graphene/spacer/graphene layer.

The presented results in Figure A1, Figure A2 and Figure A3 demonstrate a high degree of robustness against random layer-thickness variations, even for deviations as large as δ=10%. The primary effect of disorder is a smooth shift in the resonance frequency, while the resonance itself remains well defined and maintains its spectral contrast, indicating that its quality is not significantly degraded. This behavior is consistent with the topological origin of the considered TPP states. As a result, structural disorder mainly perturbs the effective phase accumulation without destroying the state, thereby allowing the power-limiting functionality of the structure to be preserved despite unavoidable fabrication imperfections.
